# Supplementary material for: Basic In-Mouth Attribute Evaluation: A Comparison of Two Panels
Source: Foods. 2018 Dec 21;8(1):3. doi: 10.3390/foods8010003 (PMC6352104; doi:10.3390/foods8010003)
Supplement: Supplementary file 1 [file foods-08-00003-s001.pdf]

Table S1. Phenolic composition of the wines

Compound (mg/L)	NEJ*CS*[a]	PV*CS*	SL*CS*	WP*CS*	NEJ*P*	NEK*P*	PV*P*	SB*P*	PV*S*	SOO*S*	WEG*S*	WER*S*
Gallic acid	33.98	63.82	39.33	44.70	26.28[b]	42.52	52.69	47.00	38.82	63.81	15.19	16.33
GRP	2.94	3.57	5.23	4.52	6.17	8.78	4.20	5.88	5.38	10.00	6.73	5.29
Delphinidin-3-glucoside	7.84	5.47	6.04	11.08	5.95	5.19	8.60	5.75	4.31	5.07	5.14	4.42
Caftaric acid	33.32	40.74	42.10	49.99	44.10	53.15	118.9	90.97	54.42	48.59	19.22	27.55
Cyanidin-3-glucoside	1.18	1.01	0.89	1.03	0.79	0.83	1.29	0.85	1.02	0.84	0.82	0.88
(+)-Catechin	27.40	45.78	43.99	30.68	15.62	22.84	38.99	23.75	31.07	40.25	13.68	14.98
Procyanidin B1	32.63	32.30	31.88	26.78	28.45	34.65	64.34	45.02	36.91	40.67	20.82	21.73
Petunidin-3-glucoside	7.15	5.44	4.97	9.69	9.72	10.11	12.39	7.77	7.83	8.58	10.08	9.31
Caffeic acid	13.04	4.18	3.66	2.73	10.79	16.34	3.51	3.65	6.15	3.64	1.24	1.53
Coutaric acid	14.78	14.35	19.10	17.11	9.77	11.87	25.04	20.96	26.18	22.57	10.47	16.31
Peonidin-3-glucoside	5.08	4.00	4.42	7.00	5.55	4.47	6.80	4.88	6.37	7.40	6.41	6.41
Malvidin-3-glucoside	43.06	57.88	39.33	61.44	64.75	68.28	93.85	48.03	66.34	61.94	73.19	75.24
Delphinidin-3-acetylglucoside	2.00	2.17	2.15	3.47	1.99	2.32	2.27	1.63	2.11	2.23	2.23	1.99

Cyanidin-3-acetylglucoside	4.34	2.50	2.00	3.34	2.98	2.44	2.16	1.68	2.58	2.58	4.42	2.55
p-Coumaric acid	5.67	4.23	2.79	1.64	3.25	5.43	2.06	1.11	3.22	4.38	2.41	2.90
Petunidin-3-acetylglucoside	1.81	1.74	1.58	2.88	1.72	2.64	2.37	2.11	1.98	2.10	2.22	2.05
Quercetin-3-glucoside	52.42	13.10	21.55	32.68	24.13	9.78	24.49	10.00	102.2	30.70	11.32	16.52
Malvidin-3-acetylglucoside	43.06	57.88	39.33	61.44	64.75	68.28	93.85	48.03	66.34	61.94	73.19	75.24
Delphinidin-3-coumarylglucoside	0.44	0.69	0.31	0.92	0.99	1.15	1.72	0.78	1.40	1.37	1.14	1.18
Petunidin-3-coumarylglucoside	1.83	1.35	1.07	1.50	1.44	1.56	2.61	1.06	2.59	2.76	2.07	2.31
Peonidin-3-coumarylglucoside	0.68	0.79	0.67	1.29	0.81	0.93	1.53	0.73	2.53	3.17	1.85	2.43
Malvidin-3-coumarylglucoside	4.28	5.89	3.62	6.17	5.51	6.62	15.45	4.62	8.11	10.37	4.53	7.69
Polymeric pigments	86.24	30.55	48.29	38.96	28.17	29.50	30.90	39.48	59.79	60.69	21.18	31.97
Polymeric phenols	1307	506	805	1011	436	433	573	737	1355	1247	289	531
Quercetin	14.28	13.21	12.48	20.92	7.24	6.63	15.66	3.62	21.29	17.01	9.61	13.26
Kaempherol	2.76	1.22	1.12	2.36	0.79	0.71	1.65	0.32	2.95	0.78	0.86	1.11

[a] Wine sample, [b] Levels of individual compounds
